# *Strongyloides* spp. in cats: a review of the literature and the first report of zoonotic *Strongyloides stercoralis* in colonic epithelial nodular hyperplasia in cats

**DOI:** 10.1186/s13071-019-3592-7

**Published:** 2019-07-12

**Authors:** Judit M. Wulcan, Michelle M. Dennis, Jennifer K. Ketzis, Thomas J. Bevelock, Guilherme G. Verocai

**Affiliations:** 1 Department of Biomedical Sciences, Ross University School of Veterinary Medicine, PO Box 334, Basseterre, Saint Kitts and Nevis; 2 Department of Infectious Diseases, College of Veterinary Medicine, University of Georgia, 501 D. W. Brooks Drive, Athens, GA 30602 USA; 3 Department of Veterinary Pathobiology, College of Veterinary Medicine and Biomedical Sciences, Texas A&M University, College Station, TX 77843 USA

**Keywords:** Colitis, Feline, *Strongyloides tumefaciens*, *Strongyloides felis*, *Strongyloides planiceps*, Pathology, Zoonosis, Strongyloidiasis

## Abstract

**Background:**

Four species of *Strongyloides*, *Strongyloides felis*, *Strongyloides planiceps*, *Strongyloides stercoralis* and *Strongyloides tumefaciens*, have been identified in cats based on morphology and location in the host with limited data on the prevalence and disease potential of these different species. *Strongyloides tumefaciens* adults are located in colonic nodules while the other three species are in the small intestine. The literature on *Strongyloides* in cats is scattered and has never been compiled. The aim of this article is to provide a short review of the existing literature on *Strongyloides* spp. in cats, to describe the pathology of colonic nodules containing *Strongyloides* sp. seen at necropsies of cats in St. Kitts, West Indies, and to provide the first unequivocal report of zoonotic *S. stercoralis* in cats based on sequencing analysis of a portion of the cytochrome *c* oxidase subunit 1 (*cox*1) gene, and supported by phylogenetic analysis.

**Results:**

Colonic nodules containing sections of nematodes, histologically compatible with *Strongyloides* sp. were seen during necropsy in six cats in St. Kitts, West Indies. Sequencing of the *cox*1 gene of the mitochondrial DNA extracted from colonic nodules from two of these cats matched sequences of the zoonotic strain of *S. stercoralis.*

**Conclusions:**

The morphological similarities between *S. stercoralis-*associated colonic nodules and previous reports of *S. tumefaciens*, together with the insufficient defining criteria for *S. tumefaciens* raises questions about the validity of the species. Further sampling and genetic characterization of isolates is needed to understand the species in cats and their zoonotic potential.

## Background

**Fig. 1:**
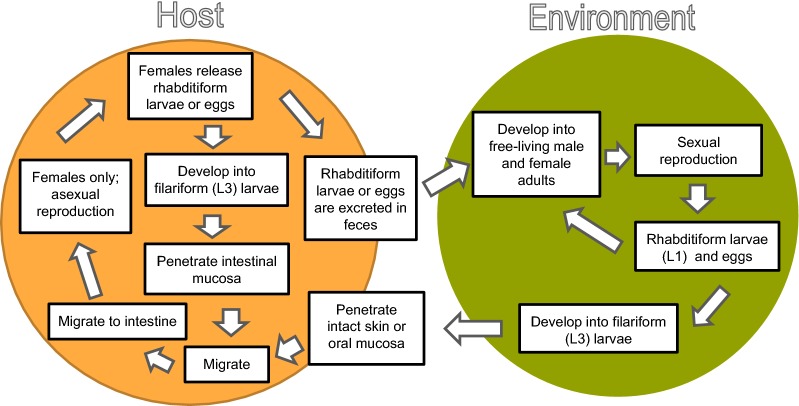
The complex free-living and parasitic life-cycle of *S. stercoralis*

*Strongyloides* (order Rhabditida, family Strongyloididae) is a genus of nematodes with a complex life-cycle that includes free-living adult stages in the environment and intestinal parasitism with female adults in a wide variety of vertebrates (Fig. 1).

*Strongyloides stercoralis* is the major *Strongyloides* species of humans and dogs and has recently been demonstrated to consist of at least two genetically isolated populations, one that is found in dogs and humans and another that has so far exclusively been demonstrated in dogs. The two different populations have not been designated as different species, but are currently referred to as different clades, one that is zoonotic and one that potentially is not [1, 2]. Prevalence estimates of human infections by *S. stercoralis* globally ranges from 30 to 100 million people, but many researchers suggest that the true prevalence could be even higher [3].

Hosts of *Strongyloides* become infected primarily through percutaneous penetration or oral ingestion of infective third-stage larvae (L3). The route of migration to the intestines is not fully understood. A pulmonary route including blood, lungs, trachea and upper gastrointestinal tract is commonly assumed for *S. stercoralis*; however, experimental infections with dogs indicate that the pulmonary route is not obligate, but rather one of many ways for the larvae to reach the intestines with larvae having limited migration and going more directly to the intestines or migrating through a variety of organs prior to reaching the intestines [4, 5]. Once in the intestines, infective L3s develop into adult females, which reproduce asexually *via* parthenogenesis. Eggs or hatched first-stage larvae (L1) are passed in the feces. The L1 can develop into a free-living adult male or female or can develop directly to an infective L3 [6]. Free-living males and females reproduce sexually with the offspring developing again into free-living males and females, depending on the species, or into infective L3 [6]. In addition to the host becoming exposed *via* infective L3 in the environment, transmammary infection can occur in some species [7]. Also, in humans and dogs, the L1 of *S. stercoralis* produced by the females in the small intestine can develop into infective L3 within the intestines resulting in “autoinfection” [6, 8, 9].

Most cases of *Strongyloides* infection in humans and animals, regardless of species, appear to be asymptomatic and self-limiting. When clinical disease is seen, it is usually in the form of acute diarrhea or bronchopneumonia in individuals with high intensity infections [10]*. Strongyloides stercoralis* infection in immunosuppressed humans and dogs can result in accelerated autoinfection leading to hyperinfection accompanied by exacerbated gastrointestinal and pulmonary signs, sometimes with evidence of disseminated infection. These types of infections are often fatal [11, 12].

*Strongyloides* spp. in cats is much less studied than *Strongyloides* in humans or other domestic animals and reports of clinical disease associated with the infection is very rare [13]. The literature on *Strongyloides* sp. infection in cats is scattered and has never been compiled. Thus, a short review of the condition follows.

Four species of *Strongyloides* have been reported in cats, namely *Strongyloides felis*, *Strongyloides planiceps*, *Strongyloides tumefaciens* and *S. stercoralis.* Species identification has been primarily based on morphological features of parasitic adult females, location in the host and eggs (Table 1) with descriptions of the L1 in feces overlapping between the species.

**Table 1:** Brief description of the *Strongyloides* stages in dogs and cats

Characteristics	*S. stercoralis*	*S. felis*	*S. tumefaciens*	*S. planiceps*
Location of parasitic female in host	Small intestine	Small intestine	Colonic nodules	Small intestine
Length of parasitic female (mm)	2–3	2.3–3.6	5^a^	2.4–3.3
Ovaries of parasitic female	Straight	Straight	Straight	Spiraled
Tail of parasitic female	Bluntly pointed	Bluntly pointed	Bluntly pointed	Bluntly rounded
Stage found in feces	Rhabditiform (L1) larvae	Rhabditiform (L1) larvae	Not indicated	Larvated eggs
Egg size (µm)	42–58 × 30–34	Similar to *S. stercoralis*	114–124 × 62–68	32–40 × 58–64
Length of L1 (µm)	180–380	217–238	200	270–520
Length of L3 (µm)	568–662	525–615	Not indicated	490–670
References	[20, 54, 55]	[14, 15]	[24]	[17]

^a^The length was estimated from pieces of two different worms dissected out from formalin embedded tissue

*Abbreviations*: L1, first-stage, rhabditiform larvae; L3, third-stage, filariform larvae

The first of the *Strongyloides* species described in cats is *S. felis* by Chandler in 1925 based on specimens from India [14]. Chandler hypothesized that it could be a subspecies of *S. stercoralis* but that until further data were available, it should be assigned its own species name. There is no further documentation of *S. felis* until 1986 when it was “rediscovered” from cats in Australia [15, 16]. Since then, there have been no studies specifically focused on the occurrence of *S. felis* or species validity.

*Strongyloides planiceps* was first isolated from the flat-headed cat or rusty tiger cat (*Prionailurus planiceps*) by R. T. Leiper in Malaysia in 1927 and was originally described by Rogers in 1939 [17]. Parasitic female *S. planiceps* differs from that of *S. felis* and *S. stercoralis* in that the ovaries are spiraled *versus* straight and the tail is bluntly-rounded *versus* bluntly-pointed (Table 1). Another distinguishing feature mentioned is that the typical fecal stage of *S. planiceps* is larvated eggs, whereas *S. felis* and *S. stercoralis* primarily shed L1 [17–19]. Sequenced *cox*1 of *S. planiceps* isolated from two Japanese raccoon dogs (*Nyctereutes procyonoides*) and *S. stercoralis* isolated from dogs supports that these are different species and indicates that *S. planiceps* and *S. stercoralis* diverged from a common ancestor prior to separation of dog-parasitic and primate-parasitic clades of *S. stercoralis* [20]. While there are many reports of *S. planiceps*, most are from wild animals and not domestic cats [18]. After the first report of *S. planiceps* in Malaysia, it has exclusively been reported from Japan where it appears to be most frequently found in raccoon dogs and Japanese weasels (*Mustela sibirica*) [18, 21, 22].

The life-cycle of *S. planiceps* and *S. felis* has been investigated and appears similar to that of *S. stercoralis*. Adult parthenogenetic females are found in the proximal small intestine and larvated eggs or L1 are passed in the feces [14, 15, 17]. One biological difference between *S. planiceps* isolated in Japan and *S. stercoralis* is that multiple generations of free-living adults can be developed experimentally with *S. planiceps*, whereas the eggs from first generation free-living *S. stercoralis* all seem to develop into filariform L3 [23].

*Strongyloides tumefaciens* was originally described by Price & Dikmans in a cat from Louisiana, USA, in 1927 and subsequently in a cat from Florida, USA, in 1930 [24]. The species description was based on morphological characteristics of incomplete parasitic females dissected from formalin-fixed paraffin-embedded colonic nodules [24]. As attempts to dissect a complete adult female from the nodules failed, parts from two different worms were pieced together to estimate the length for the adult nematode (approximately 5 mm). Apart from the size, morphological features for the parasitic female were similar to those previously described for *S. felis* and *S. stercoralis*. A new species, *S. tumefaciens*, was proposed based on the longer size of the adult female and its location in the colon (*versus* small intestine) [24]. Since then there have been infrequent reports in the USA (1977 and 1987) [25, 26], Brazil (2012 and 2013) [27] and India (1964) [13]. In all cases, female *S. tumefaciens* have only been observed within nodular lesions in the colonic wall; no adults in the lumen or free-living adults have been observed [24–27]. The pathology of *S. tumefaciens*-associated colonic nodules has been similar in all reports from domestic cats [24–27] (Table 2). Multiple 2–20 mm in diameter white submucosal colonic nodules, most apparent from the mucosal surface, that often have a central punctuate depression have been observed in all affected cats. Histologically, the nodules consist of nodular (sometimes referred to as adenomatous) hyperplasia of colonic epithelial cells. The epithelial cells form tubules that extend into the submucosa, are supported by delicate stroma heavily populated by lymphoid cells and contained within a fibrous capsule. Cross-sections of parasitic female nematodes and numerous larvae are abundant throughout the tubules and stroma. In two cases, small numbers of *Strongyloides* larvae were identified upon fecal examination or within the intestinal lumen [25, 26]. Clinical data for *S. tumefaciens* are limited by the fact that the diagnosis has never been made *ante mortem*.

**Table 2:** Signalment, history and lesions in cases of *S. tumefaciens* reported in domestic cats

Location, year	Signalment	History	Size of colonic nodules (mm)	Other lesions	Reference
Louisiana, 1927	Domestic cat	Died	2–10	ni	[24]
Florida, 1930	Domestic cat	Died after period of chronic diarrhea	2–10	ni	[24]
Louisiana, 1977	6 months, intact female, Domestic Shorthair (DSH) cat	Died after period of anorexia and serous oculonasal discharge. Recent fecal examination with small numbers of *Strongyloides* larvae, *A. tubaeforme* and *T. cati* eggs	2–3	Pleuritis	[25]
Georgia, 1987	Male, Siamese	Euthanized due to suspicion of feline leukemia	2–3	ni	[26]
Brazil, 2012	Adult, male, mongrel domestic cat	Died after period of debilitation, muscle weakness and icterus	2–20	Jaundice; focally extensive necrosuppurative hepatitis; splenomegaly; lymphadenomegaly	[27]
Brazil, 2013	Adult, male, mongrel domestic cat	Died after period of chronic diarrhea and progressive weight loss	2–20	None	[27]

*Abbreviations*: ni, not indicated

It is challenging to determine when *S. stercoralis* was first reported in cats. While it had been suggested that *S. felis* could be a subspecies of *S. stercoralis* [14], attempts to experimentally induce infections in cats with *S. stercoralis* from dogs and humans resulted in relatively light infections that cleared quickly [28, 29]. For this reason, *S. stercoralis* was not considered a normal parasite of cats and the assignment of *S. felis* as the species of cats remained accepted by many in the field. However, in the last 11 years, there have been reports of *S. stercoralis* in cats from Qatar, Brazil and Kenya [30–32]. These reports do not highlight that *S. felis* and not *S. stercoralis* was previously believed to occur in cats and the studies, which all lack molecular confirmation of species identification, provide little information regarding how the species was identified.

The true prevalence and geographical distribution of *Strongyloides* spp. in cats is largely unknown, with most studies reporting the species present based on assumptions regarding the original geographical location of each species. Most cat parasite prevalence studies utilize fecal samples collected at a single time-point and processed using flotation methods, which are known to have a low sensitivity for detecting *Strongyloides* larvae. The Baermann technique and its modifications were utilized to increase larval detection in only one of the surveys identified in the literature [16]. Prevalence studies, in which *Strongyloides* spp. have been diagnosed in cats are summarized in Table 3.

**Table 3:** Studies in which *Strongyloides* spp. in cats were demonstrated in some nations

Location	*n*	Prevalence (%)	Sampling method	Examination method	Species	Reference
Australia	504	33.5	Necropsy	Baermann	*S. felis*	[16]
Australia	28	46.4	Necropsy	Flotation	*Strongyloides* sp.	[44]
Brazil	37	54.1	Environmental collection	Flotation	*Strongyloides* sp.	[39]
Brazil	173	13.9	Environmental collection	Flotation	*S. stercoralis* ^a^	[31]
Denmark	99	1.0	Necropsy	Sedimentation and counting	*Strongyloides* sp.	[41]
England	131	1.5	ni	Flotation	*Strongyloides* sp.	[40]
India	ni	20	ni	ni	*S. felis*	[14]
Japan	105	3.8	Necropsy	Flotation	*S. planiceps*	[22]
Kenya	103	43.7	Litter box collection	Formalin-ether sedimentation	*S. stercoralis* ^a^	[32]
Qatar	824	18.4	Environmental collection	Formalin-ether sedimentation	*S. stercoralis* ^a^	[30]
Romania	414	3.4	Litter box collection	Flotation	*Strongyloides* sp.	[43]
Thailand	300	0.7	ni	Formalin-ether sedimentation	*Strongyloides* sp.	[42]

^a^Species identification method unclear

*Abbreviations*: ni, not indicated

## Methods

### Literature review

A literature review utilizing the search terms (*Strongyloides* OR Threadworms) AND (cats OR felines), was performed in PubMed [33] on September 27, 2018. Out of 46 identified records, 17 remained after title and abstract screening. Fourteen additional records were identified from review articles, book chapters and unstructured google searches. In total 6 review articles [10, 34–38], 6 experimental studies [19, 21, 23, 28, 29], 13 surveys [14, 16, 18, 22, 30–32, 39–44] and 6 case reports [15, 17, 24–27] were reviewed.

### Selection of cases

Fourteen St. Kitts cats euthanized due to FIV, ill health or accidents between February 2013 and March 2014, underwent necropsy for nematode collection at the Ross University School of Veterinary Medicine, St. Kitts, West Indies. Most of the cats (12/14) were feral cats that had been trapped for spay, neuter and release but were euthanized due to FIV positive status or other health concerns.

Thirty cats, submitted for necropsy from the clinic at the Ross University School of Veterinary Medicine or collected road-killed stray cats, were examined for the presence of colonic nodules between January and July 2018. These cats were examined with the specific objective to evaluate whether large intestinal nodules were associated with *Trichuris* sp. infections in cats.

### Histopathology

Grossly visible colonic nodules were collected from all cats. Tissues were fixed in 10% neutral-buffered formalin, routinely processed and embedded in paraffin wax. Sections cut 4-µm thick were stained with hematoxylin and eosin.

### Immunohistochemistry

Colonic sections from four cats with *Strongyloides*-associated nodules and four cats with colonic nodules consisting of GALT, were evaluated immunohistochemically to detect CD3 (cells of T-cell origin) and CD79a (B-cells).

Immunohistochemistry using polyclonal rabbit anti-human CD3 (DAKO, 1:400) was performed on a Ventana Benchmark XT platform (Ventana Medical Systems Inc., Tucson, AZ, USA) using an anti-rabbit/anti-mouse alkaline phosphatase-labelled polymer detection kit (Ventana Medical Systems Inc., Tucson, AZ, USA) with UltraVIEW Red chromogen and Harrisʼs hematoxylin counterstain (Ventana Medical Systems Inc., Tucson, AZ, USA).

Immunohistochemistry for monoclonal (HM57) mouse anti-human CD79a (DAKO 1:200) was performed on a DAKO autostainer platform (Dako Canada Inc., Burlington, ON, Canada) using a goat anti-mouse horseradish peroxidase-labelled polymer detection kit (UltraVisionONE, Lab Vision Corp., Fremont, CA, USA) with Nova Red chromogen (Vector Laboratories, Burlington, ON, Canada) and Harrisʼs hematoxylin counterstain.

Antigen retrieval was achieved by heat induced epitope retrieval at pH 8 on the Ventana Benchmark XT platform for CD3, and by heat induced epitope retrieval at pH9 on a Decloaking Chamber from BioCare Medical (BioCare Medical, Concord, CA, USA) for CD79a.

Negative controls were made by substituting non-immune rabbit serum (for CD3) or antibody diluent (for CD79a) for the primary antibody. Feline lymph node was used as a positive control.

### Molecular analysis

Extraction of genomic DNA from paraffin-embedded colonic nodules was attempted for 5/6 cats with *Strongyloides* associated lesions. One cat, with a lesion histologically categorized as a *Strongyloides* sp. associated colonic nodule was identified after the analysis was performed, and not included. DNA was extracted using a QIAmp DNA FFPE tissue kit following manufacturer’s protocol (Qiagen, Valencia, CA, USA).

DNA templates were subjected to a polymerase chain reaction (PCR) for targeting a fragment of the cytochrome *c* oxidase subunit 1 (*cox*1) gene of the mitochondrial DNA (mtDNA) using primers TJ5207 (5′-TTT GAT TGT TAC CTG CTT CTA TTT T-3′) and TJ5208 (5′-TTT TAC ACC AGT AGG AAC AGC AA-3′) [1]. PCR was performed in 25 µl reactions including Go^™^Taq Green Master Mix (Promega, Madison, WI, USA), 0.5 µmol/l of each primer and 5 µl of DNA template. PCR conditions followed Jaleta et al. [1] and included: denaturation at 94 °C for 2 min, followed by 35 cycles of 94 °C for 30 s, 50 °C for 15 s, and 72 °C for 90 s, with a final 10 min extension at 72 °C. Successful amplification was confirmed by visualization of bands of approximately 650 bp using gel electrophoresis. PCR products were purified using the E.Z.N.A. Cycle Pure kit (Omega Bio-Tek, Norcross, GA, USA) and sequenced in both directions using the forward and reserves PCR primers using BigDye Terminator Cycle Sequencing (Applied Biosystems, Foster city, CA, USA).

### Phylogenetic analysis

Fragments of the *cox*1 gene from two cats were successfully sequenced, edited and aligned in MEGA v.7 [45]. Phylogenetic analysis was performed using MEGA v.7 [45] using the Maximum Likelihood method with 1000 bootstrap replicates. The best-fitting evolutionary model for the data set was General Time Reversible, Gamma distributed (GTR+G). All sequences were trimmed to 522 bp. Homologous sequences of isolates of *S. stercoralis* from humans, dogs and non-human primates, as well as those of other *Strongyloides* species, and *Necator americanus* (outgroup) were included in the analysis.

### Total worm counts

For the cats collected in 2013–2014, the small and large intestine, after sections for histopathology were collected, were opened, soaked in saline at approximately 36 °C for 1 to 3 h and then scraped and washed over a 50 μm sieve. Sieved content was examined for nematodes, with an Olympus SZX16, magnification of 20–100×.

For the cats collected in 2018, the remainder of the whole gastrointestinal tract, divided into stomach, small intestine and large intestine, along with any content was soaked in saline at approximately 36 °C for 3–24 h and then scraped and washed over a 100 μm sieve. Sieved content was fixed in 5–10% formalin and stored together with the formalin used for section fixation. The stored small and large intestinal content was examined for nematode stages with an Olympus SZX16 at 25–100× magnification. The person responsible for assessing worm counts was blinded to the intestinal pathology results.

### Statistics

This is a descriptive study. Findings that can be tested statistically (possible associations between number of colonic nodules, size of colonic nodules and CD3/CD79a staining, with *Strongyloides* infection status) should be investigated in a hypothesis driven study, with appropriate (pre-determined) sample size to avoid spurious results.

## Results

**Fig. 2:**
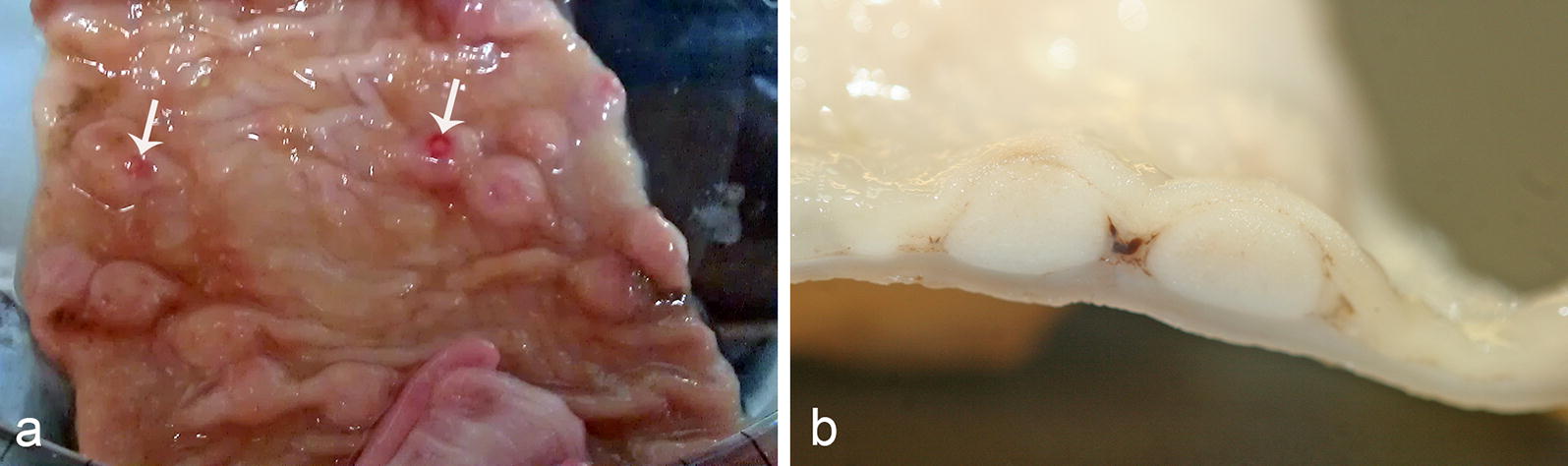
*Strongyloides*-associated colonic nodules, cat. **a** Mucosal surface, colon cat. The nodules are protruding into the mucosal lumen. Small central depressions are visible on the mucosal surface of the nodules (arrows). **b** Cut surface of colonic nodules

**Fig. 3:**
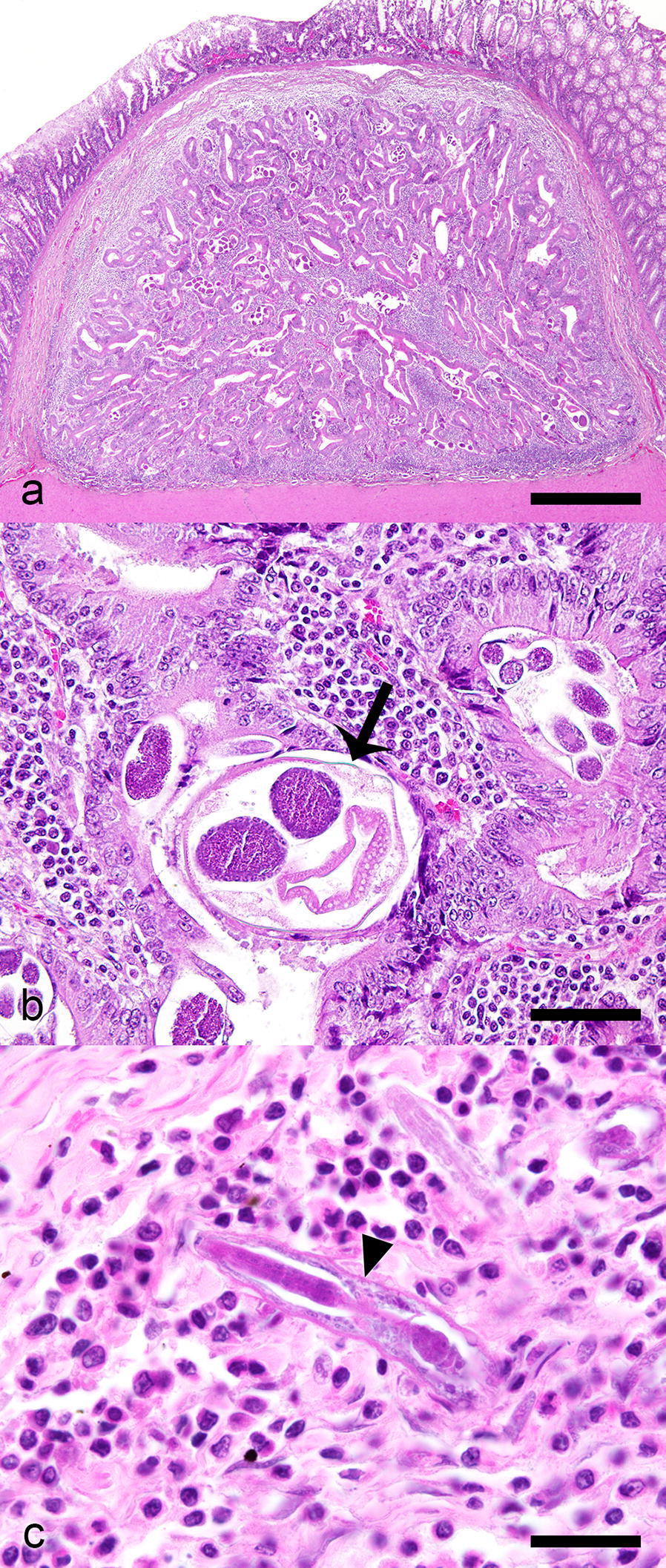
*Strongyloides* colonic epithelial nodular hyperplasia, cat. Hematoxylin and eosin. **a** Encapsulated nodule of hyperplastic colonic epithelial cells which form branching tubules, surrounded by a predominately lymphoplasmacytic infiltrate within the submucosa. **b** Adult nematodes (arrow) are within tubules. **c** Rhabditiform larvae (arrowhead) within the nodule stroma surrounded by lymphocytes and plasma cells. *Scale-bars*: **a** 250 μm; **b** 70 μm; **c** 20 μm

*Strongyloides* sp. were diagnosed from the postmortem examination of six cats at Ross University School of Veterinary Medicine, St. Kitts, West Indies. For three of these cats, postmortem examinations were performed for collection of specimens of *Trichuris* sp. between February 2013 and March 2014 (*n* = 14); and postmortem examinations were performed on the other three cats between January and July 2018, for a study investigating a potential association between large intestinal pathology and *Trichuris* infection status (*n* = 30). No *Strongyloides* spp. stages were identified in total worm counts on any of the cats. Grossly, multifocal, slightly raised, off-white nodules, 1–8 mm in diameter, were observed in the colonic wall of *Strongyloides* infected cats. The largest nodules were visible from both the mucosal and serosal surfaces, had small pits or depressions on their mucosal surface, and appeared on cut surface to be located in the submucosa (Fig. 2). Microscopically, these nodules consisted of colonic epithelial nodular hyperplasia and colitis containing *Strongyloides* sp. The nodules were composed of tubules of hyperplastic mucosal epithelial cells, supported by scant fibrous stroma, and containing aggregates of lymphocytes and fewer plasma cells, in some cases mixed with eosinophils, resembling gut associated lymphoid tissue (GALT) (Fig. 3). *Strongyloides* larvae, adult nematodes, and eggs were within tubules and stroma (Fig. 3). In every case, the diagnosis of *Strongyloides* sp. was based on morphological features of the parasite in tissue section: adult parasitic females were approximately 110 µm in diameter, had platymyarian musculature, an intestine composed of uninucleate cells, and paired genital tracts.

**Fig. 4:**
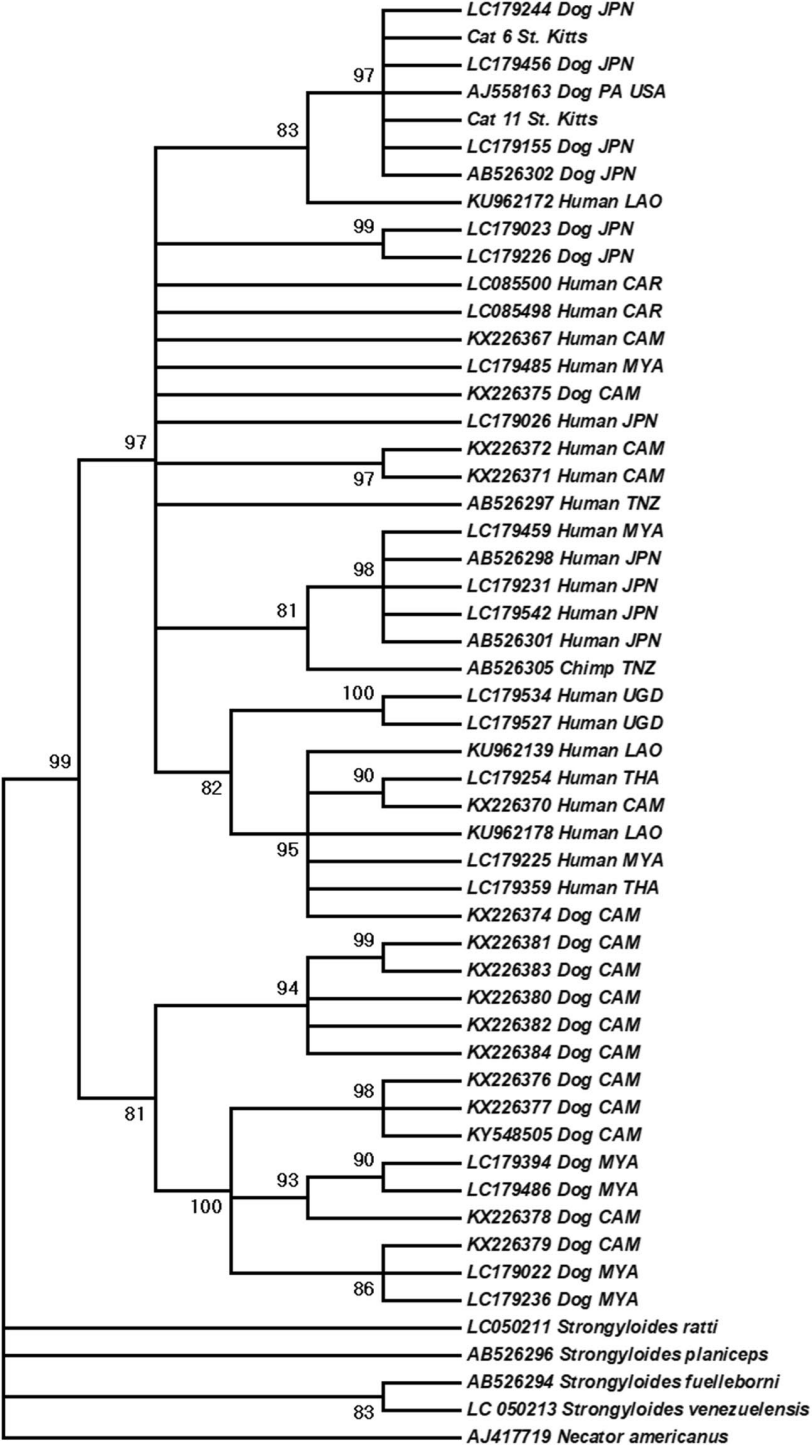
Maximum likelihood phylogenetic tree depicting the relationship of *Strongyloides stercoralis* isolates from cats from Saint Kitts (“cat 6 St. Kitts”, GenBank accession number MK463927 and “cat 11 St. Kitts”, GenBank accession number MK463928), and isolates from different hosts and geographical origins, based on a fragment of the cytochrome *c* oxidase subunit 1 of the mitochondrial DNA (1000 bootstrap replicates). Numbers at nodes indicate bootstrap values, nodes with less than 80% support were collapsed

Fragments of the *cox*1 gene were successfully sequenced from two samples of two affected cats and were subjected to phylogenetic analysis. In three other affected cats, attempts to extract large enough fragments of DNA for the PCR to work was unsuccessful, most likely due to DNA degradation secondary to FFPE preservation. The two sequences generated belong to *S. stercoralis* and were identical to each other (GenBank accession numbers: MK463927 and MK463928 [46]), and the pairwise distance between these isolates and other *S. stercoralis* isolates included in the analysis ranged between 0–0.06. All *S. stercoralis* sequences formed a well-supported clade (99% bootstrap support). This clade was divided into two well-supported clades. The St. Kitts’ cat samples clustered with isolates assumed to be zoonotic and associated to dogs, humans and non-human primates from North America, Asia and Africa (97% bootstrap), and the other clade comprised only dog isolate from East and Southeast Asia (81% bootstrap) (Fig. 4).

Grossly the *Strongyloides* sp. associated colonic nodules resembled GALT which was seen in 14 of the 30 cats examined in 2018 and nine of the 14 cats examined in 2013 and 2014. Smaller accumulations of GALT that were not grossly evident were also in the colonic submucosa of all cats.

**Fig. 5:**
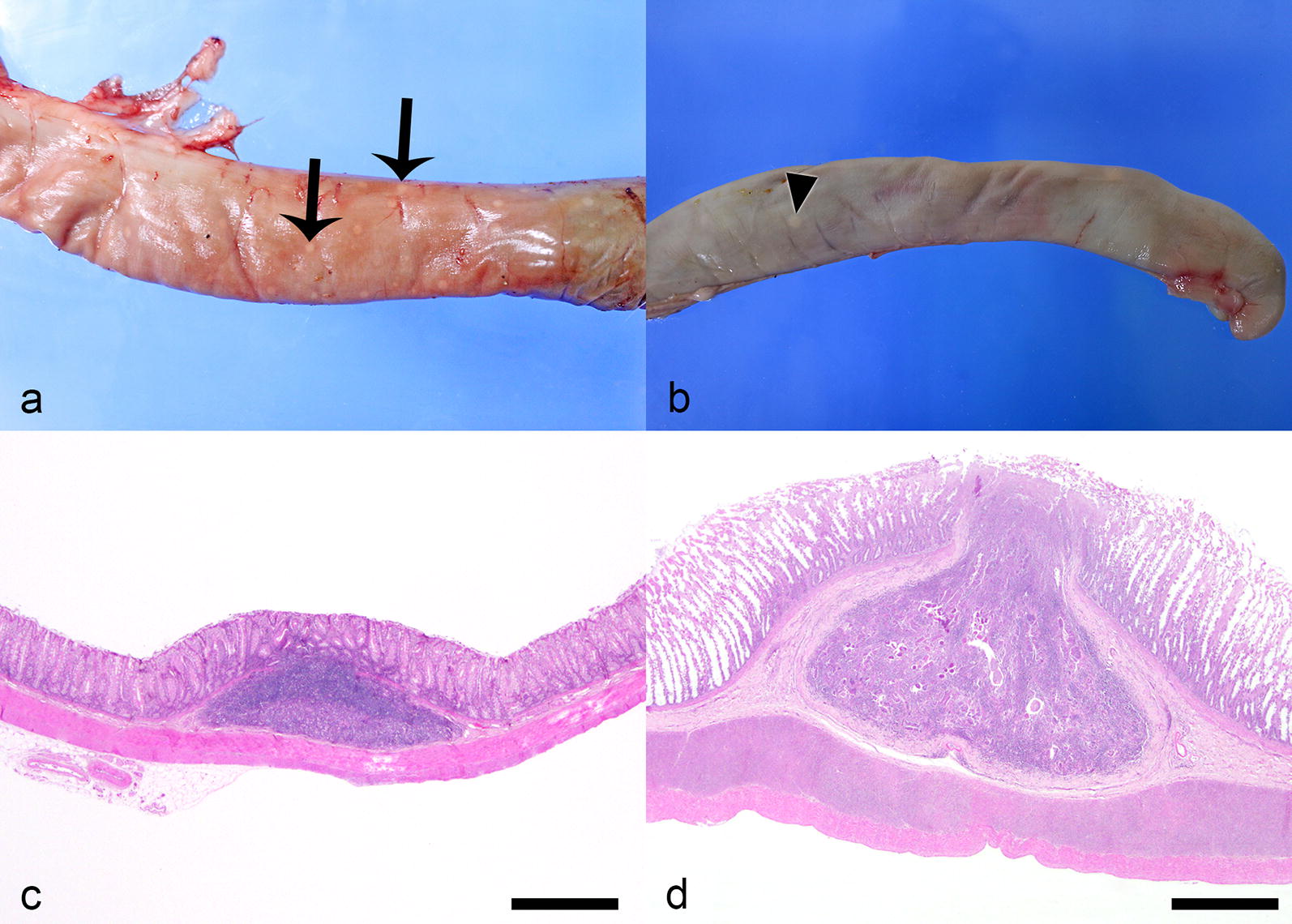
Comparison of GALT and *Strongyloides* colonic epithelial nodular hyperplasia, cat. **a** GALT (arrows) evident from the colonic serosal surface. **b** A single focus of *Strongyloides* colonic epithelial nodular hyperplasia (arrowhead) evident from the colonic serosal surface. **c** Colonic GALT consists of well-demarcated collection of lymphoid tissue within the colonic submucosa. Hematoxylin and eosin. **d** *Strongyloides* colonic epithelial nodular hyperplasia similarly consists of well-demarcated aggregate of lymphocytes and plasma cells, but also contains nematodes and herniated tubules of hyperplastic colonic epithelial cells. Hematoxylin and eosin. *Scale-bars*: **c**, **d** 500 µm

To evaluate potential distinguishing factors between *Strongyloides* colonic epithelial nodular hyperplasia and GALT, the size and number, gross appearance, and lymphoid composition of colonic nodules was compared. The number and size of colonic nodules in the 12 cats collected in 2013–2014 was not recorded. For the cats collected in 2018, colonic nodules histologically consisting entirely of GALT were all between 1–2 mm in diameter, whereas *Strongyloides*-associated nodules were larger, ranging from 2–8 mm in diameter (median 4 mm). The median number of grossly visible colonic GALT nodules in each cat was 25 (range 5–120), whereas the median number of *Strongyloides*-associated colonic nodules in each cat was 2 (range 1–15). Mucosal depressions were seen in the *Strongyloides*-associated nodules (Fig. 5) and the GALT nodules.

**Fig. 6:**
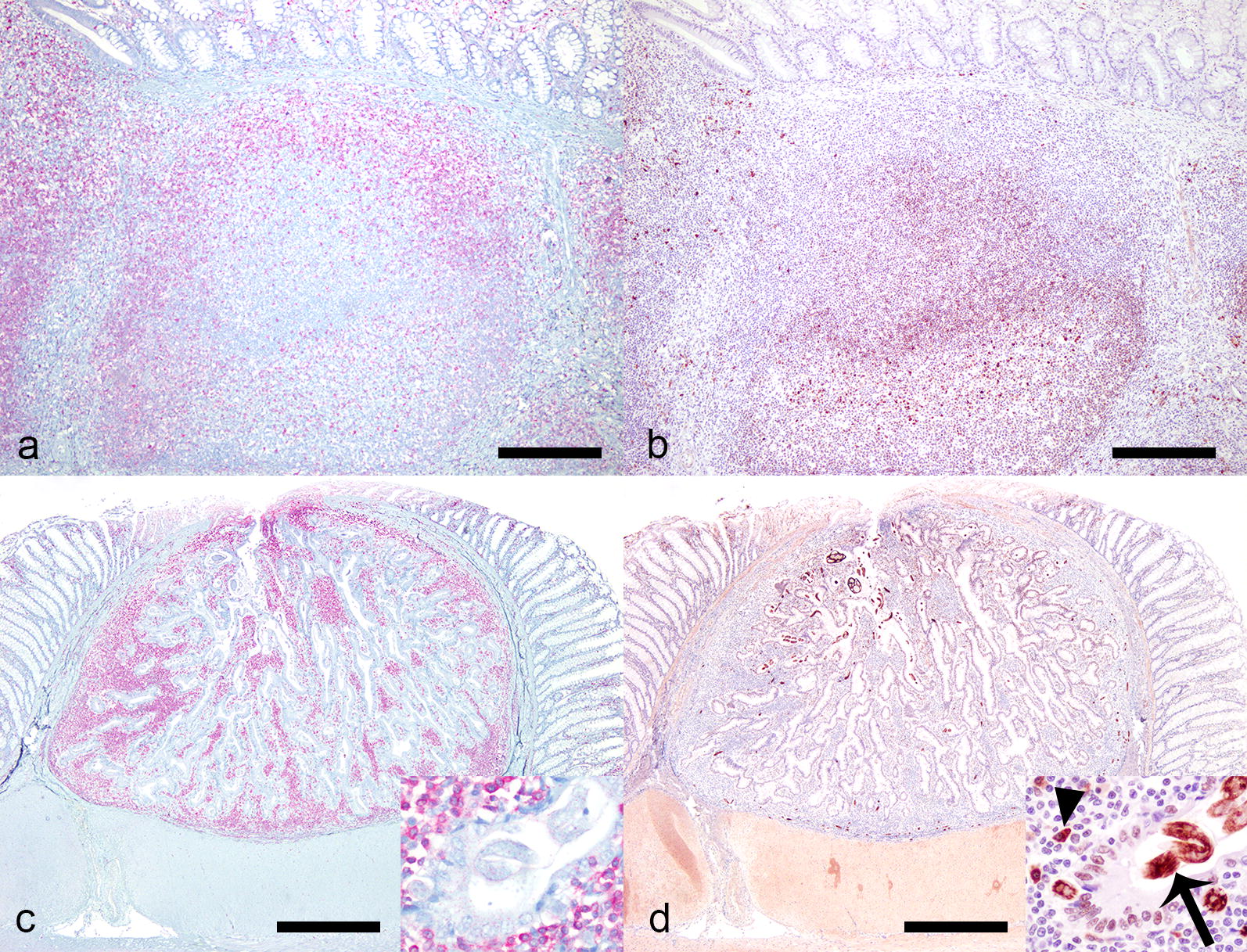
Comparison of lymphocyte infiltrate in GALT and *Strongyloides* colonic epithelial nodular hyperplasia, cat. **a**, **b** GALT, colon, cat. **a** There is an unevenly distributed population of T-cells positive for CD3 (Immunohistochemistry for CD3). **b** There is a similar uneven distribution of B-cells positive for CD79a (Immunohistochemistry for CD79a). **c**, **d** *Strongyloides* colonic epithelial nodular hyperplasia. **c** The majority are T-cells positive for CD3 (inset) (Immunohistochemistry for CD3). **d** Nematodes (inset, arrow) and low numbers of plasma cells (inset arrowhead) are positive for CD79 (Immunohistochemistry for CD79a). *Scale-bars*: **a**, **b** 250 µm; **c**, **d** 300 µm

The colonic sections submitted for CD3/CD79a (T/B lymphocyte) immunohistochemistry represented four *Strongyloides*-affected cats, and four non-affected cats, and contained in total 17 *Strongyloides*-associated nodules and 48 foci of submucosal or mucosal GALT. GALT consistently (48/48) was composed of a heterogeneous population of lymphocytes showing cytoplasmic immunoreactivity for CD3 (T-cells) and CD79a (B-cells). All *Strongyloides*-associated nodules (17/17) consisted predominantly of lymphocytes that stained positive for CD3 (T-cells), and much fewer plasma cells that stained positive for CD79a (B-cells). The sections of *Strongyloides* stages themselves all stained strongly positive for CD79a (Fig. 6).

## Discussion

The isolation of the zoonotic strain of *S. stercoralis* from colonic nodules, morphologically similar to lesions previously attributed to *S. tumefaciens*, is the first unequivocal report of *S. stercoralis* from naturally infected cats. Moreover, it also raises questions regarding the validity of *S. tumefaciens* as a separate species. Morphological differences reported between *S. stercoralis*, *S. tumefaciens* and *S. felis* are limited to morphometric differences determined from only a few observations of often incomplete specimens, specifically in the case of *S. tumefaciens*.

The true diversity of *Strongyloides* species infecting domestic cats, the true host range, and the validity of some of the species, needs to be reassessed by means of targeted epidemiological studies using integrated diagnostic approaches which includes the use of the Baermann technique for larval isolation and molecular methods, such as sequencing and phylogenetic analysis. Systematical descriptions of specimens collected in such studies could be used to determine inter- and intraspecies variations in morphological features. To determine potential differences in the life-cycle between different species and hosts, culture of free-living stages and experimental infections of laboratory hosts would be beneficial.

It would be of particular benefit to molecularly characterize nematodes retrieved from cats in the southern USA from where *S. tumefaciens* was originally described and nematodes from cats in Australia, where a high prevalence of *S. felis* has been reported. The pathological presentation of the infection in colonic nodules in cats might provide valuable insight in *S. stercoralis* pathogenesis. The location of the worms, within colonic nodules, is not typical in most *Strongyloides* species; however, it is not unheard of in human *S. stercoralis* infection. In a retrospective case-series from 2011 on 10 human patients with *S. stercoralis* infections, yellowish-white nodules were observed in the colon. Biopsies of the nodules revealed *Strongyloides* filariform larvae [47].

The *S. stercoralis*-associated colonic nodules seen in the study presented herein were not always easy to discern on gross examination from isolated lymphoid nodules, a normal component in the colonic wall. Although all of them were grossly visible, the smaller lesions were less visible from the mucosal surface and were especially similar to GALT. It is therefore likely that these lesions might be more common than they are reported. One possibility is that colonic nodules are a common, but notoriously overlooked feature of *S. stercoralis* infections in general. Another possibility is that it is a reaction to an infection in an unusual, less suitable, host.

The colonic nodules within which the parasites are found have previously been described as primarily epithelial hyperplastic or adenomatous lesions with an inflammatory (predominantly lymphocytic) infiltrate [24–27]. One study suggests the possibility that the lymphoid tissue is remnants of lymphoid clusters (GALT) [27]. GALT includes Peyerʼs patches in the small intestine and isolated lymphoid follicles throughout the whole gastrointestinal tract. They are part of the afferent or inductive compartment of the gastrointestinal immune system, which function to prime naive T and B cells to initiate the immune response, in response to local antigens. Both Peyerʼs patches and isolated lymphoid follicles consists of a combination of B-cells and T-cells [48]. All GALT examined in this study consisted of a combination of cells that stained positive for CD3 (T-cells) and cells that stained positive for CD79 (B-cells), whereas the majority of the cells in all *Strongyloides-*associated nodules examined stained positive with CD3 (T-cells). This may indicate that the *Strongyloides-*associated nodules are part of an efferent T-cell dominated immune response against the nematodes, rather than herniation into preexisting GALT. Another possibility is that the presence of nematodes within GALT downregulates signals for B-cells which changes the composition of the nodules. This hypothesis is highly speculative as similar mechanisms have not been described previously for *Strongyloides* spp. There are, however, studies on other parasites where the parasites have been shown to produce proteins that interact with human B-lymphocytes and potentially play a role in suppression of molecules that determine the activation of B-lymphocytes [49]. The strong CD79 staining of intranodular *Strongyloides* stages observed may be supportive of a similar mechanism for *S. stercoralis*.

The study reported here from St. Kitts was neither designed to report the prevalence of *Strongyloides* sp. in cats of St. Kitts nor to evaluate potential risk factors for infection in cats. However, *S. stercoralis* in St. Kitts’ cats is likely not uncommon given the number seen when one specifically looks for them. No *Strongyloides* stages were, however, observed in any of the total worm counts performed in this study nor have any *Strongyloides* stages been observed in over 200 fecal flotation analyses performed with feral cats over the last three years. In addition, in a study by Krecek et al. [50] in which several methods were used including Baermann examination, no *Strongyloides* stages were seen in approximately 100 samples from feral cats from St. Kitts. One explanation could be that fecal excretion is not a common feature of *S. stercoralis* in cats. Alternatively, fecal excretion of eggs and L1 is low and the methods used, or quantity of feces analyzed has been insufficient to detect the infections. There are limited data on the prevalence of *S. stercoralis* in humans on St. Kitts, but the prevalence has been estimated to be below 1% [51]. As with cats, routine fecal analysis for dogs at the RUSVM diagnostic laboratory does not include the Baermann technique and no reports of *Strongyloides* could be found in recent years with older data not accessible. To further investigate the host range of *S. stercoralis* encountered in St. Kitts’ cats, future prevalence studies could in addition to cats, include humans, dogs and potentially also introduced wildlife, such as wild carnivores (Indian mongoose, *Herpestes auropunctatus*) and primates (African green monkey, *Chlorocebus aethiops sabaeus*). In future prevalence studies, the Baermann technique, serial analysis and PCR should be utilized.

On St. Kitts, although there is no feline leukemia virus (FeLV), the prevalence of feline immunodeficiency virus (FIV) is relatively high in the cat population with estimates ranging between 10–30% [52, 53]. The frequent observations of *Strongyloides* in an area high in FIV might be mere coincidence, but as *S. stercoralis* infection is associated with immunosuppression in humans, a possible association should be evaluated in future studies.

## Conclusions

To our knowledge, this is the first unequivocal report of natural zoonotic *S. stercoralis* infection in cats. The location of *S. stercoralis*, within colonic nodules consisting of epithelial hyperplasia and cells staining positive for CD3 (T-cells), and the CD79a staining of the intranodular *Strongyloides* stages observed, may provide new insight to the pathogenesis of this infection. The colonic nodules presented here are morphologically similar to those previously described for *S. tumefaciens* for which there are insufficient defining criteria, hence raising questions about its validity as a species. Further sampling and genetic characterization of isolates are needed to better define which *Strongyloides* species infect cats and their zoonotic potential.

## Data Availability

The nucleotide sequences analyzed during the present study are available in the GenBank database under the accession numbers MK463927 and MK463928. Additional datasets used and/or analyzed are available from the corresponding author upon reasonable request.

## Abbreviations

*cox*1: cytochrome *c* oxidase subunit 1
FeLV: feline leukemia virus
FIV: feline immunodeficiency virus
GALT: gut associated lymphoid tissue
GSVP: Georgia Veterinary Scholar Summer Programme
L1: first-stage larvae
L3: third-stage larvae
mtDNA: mitochondrial DNA
PCR: polymerase chain reaction
